# Objective Physical Function in the Alzheimer’s Disease Continuum: Association with Cerebrospinal Fluid Biomarkers in the ALBION Study

**DOI:** 10.3390/ijms241814079

**Published:** 2023-09-14

**Authors:** Stefanos N. Sampatakakis, Eirini Mamalaki, Eva Ntanasi, Faidra Kalligerou, Ioannis Liampas, Mary Yannakoulia, Antonios N. Gargalionis, Nikolaos Scarmeas

**Affiliations:** 1 1st Department of Neurology, Aiginition Hospital, National and Kapodistrian University of Athens Medical School, 11528 Athens, Greece; stefanos.sab@gmail.com (S.N.S.); eir.mamalaki@gmail.com (E.M.); e.ntanasi@hotmail.com (E.N.); fkaliger@gmail.com (F.K.); 2 Department of Neurology, University Hospital of Larissa, School of Medicine, University of Thessaly, 41100 Larissa, Greece; liampasioannes@gmail.com; 3 Department of Nutrition and Diatetics, Harokopio University, 17671 Athens, Greece; myianna@hua.gr; 4 Department of Medical Biopathology and Clinical Microbiology, Aiginition Hospital, National and Kapodistrian University of Athens Medical School, 11528 Athens, Greece; agargal@med.uoa.gr; 5 The Gertrude H. Sergievsky Center, Taub Institute for Research in Alzheimer’s Disease and the Aging Brain, Department of Neurology, Columbia University, New York, NY 10032, USA

**Keywords:** walking time, Alzheimer’s Disease continuum, cerebrospinal fluid biomarkers, amyloid-β 42

## Abstract

Cognitive and physical decline, both indicators of aging, seem to be associated with each other. The aim of the present study was to investigate whether physical function parameters (walking time and handgrip strength) are related to cerebrospinal fluid (CSF) biomarkers (amyloid-beta Aβ_42_, Tau, PhTau) in individuals in the Alzheimer’s disease (AD) continuum. The sample was drawn from the Aiginition Longitudinal Biomarker Investigation of Neurodegeneration study, comprising 163 individuals aged 40–75 years: 112 cognitively normal (CN) and 51 with mild cognitive impairment (MCI). Physical function parameters were measured at baseline, a lumbar puncture was performed the same day and CSF biomarkers were analyzed using automated methods. The association between walking time, handgrip strength and CSF biomarkers was evaluated by linear correlation, followed by multivariate linear regression models adjusted for age, sex, education and APOEe4 genotype. Walking time was inversely related to CSF Aβ_42_ (lower CSF values correspond to increased brain deposition) in all participants (*p* < 0.05). Subgroup analysis showed that this association was stronger in individuals with MCI and participants older than 60 years old, a result which remained statistically significant after adjustment for the aforementioned confounding factors. These findings may open new perspectives regarding the role of mobility in the AD continuum.

## 1. Introduction

Alzheimer’s Disease (AD), the most common cause of dementia, has been recognized as a multifaceted process along a cognitive and biological continuum [1]. Beginning from preclinical stages with normal cognition although biochemical evidence of neurodegeneration may exist, the disease progresses to its clinical stages, including mild cognitive impairment (MCI) and AD dementia [2]. Neuropathological changes in AD precede clinical manifestations [3], thus several biomarkers of the AD continuum have been recognized, such as cerebrospinal fluid amyloid beta (CSF Aβ_42_) [4,5], Tau (CSF Tau) [6] and Phosphorylated Tau (CSF PhTau), which may be present in preclinical stages, as well.

Aging is the major risk factor of AD and cognitive decline [7]. Physical decline depicts an aspect of aging as well, [8] which is common in the elderly and leading to an increased susceptibility to adverse health outcomes [9]. To date, physical decline has been studied in the context of gait disturbances and musculoskeletal changes. Among different gait parameters, gait speed, which is simple and representative, is commonly used for investigating gait performance in the elderly [10]. At the same time, previous studies suggested that handgrip strength, which is easy to measure, may be used as a reliable and useful index of total upper extremity muscle strength and overall physical status [11].

Much evidence shows that physical and cognitive function are associated with each other [12]. A low level of physical activity has been reported as a potential risk factor for dementia [13], while physical activity seems to improve cognition in MCI and dementia [14]. Gait disturbances have been shown to occur in relatively early stages of the progression of AD [15,16,17]. Muscle weakness is more frequent in persons with AD dementia [18], while several studies have reported that poor handgrip strength is associated with a greater risk of cognitive impairment [11,19]. The underlying mechanisms of these relationships still need to be clarified [20].

To our knowledge, limited data exist regarding the relation between physical function parameters and biomarkers of the AD continuum. There are only a few relevant studies of CSF biomarkers [21,22,23,24] which showed no significant results, presenting remarkable limitations. Most studies include only individuals with dementia and a low number of participants (<100), limiting the studies’ power. A time interval between walking time and biomarkers’ measurements exists in several studies, while the increased heterogeneity of results has been observed in multicenter studies.

Thus, our aim was to expand knowledge regarding the physical function parameters in the AD cognitive and biological continuum by presenting data from a study investigating the biomarkers of neurodegeneration. For that purpose, we conducted specific analysis in which we examined the association of both grip strength and walking time (an equivalent of gait speed) with all relevant CSF biomarkers using a biomarker automated method (Roche Diagnostics) in individuals of a broader age range (40–75 years old) belonging in the AD continuum.

## 2. Results

### 2.1. Baseline Demographic and Clinical Characteristics

In total, 163 individuals of ALBION’s first evaluation were included in our analysis. Their main characteristics are presented in Table 1. Regarding demographic data, there was a female predominance in this cohort. More than two-thirds (68.7%) of the participants were classified as cognitively normal (CN). Analysis between participants with normal cognitive function and participants with MCI showed that family history of dementia was similar between the two groups. As expected, age differed between the two different clinical groups of the AD continuum, as participants with MCI were older. Cognitive scores (MMSE and ACE) were lower in the MCI group (*p* < 0.001), while physical function parameters were statistically different according to clinical diagnosis [walking time was higher (*p* < 0.001) and grip strength was lower (*p* = 0.032) in MCI individuals] as well. Regarding CSF biomarkers, CSF Aβ_42_ was lower (*p* = 0.005) and CSF PhTau was higher (*p* < 0.001) in the MCI group, whereas CSF Tau did not differ between the groups (*p* = 0.171).

### 2.2. CSF Biomarkers and Physical Function Parameters in Different Groups

Subgroup analysis of CSF biomarkers and physical function parameters was performed, as shown in Table 2. Concerning CSF biomarkers, CSF Aβ_42_ decreased with age (*p* = 0.007). In contrast, CSF Tau (*p* = 0.009) and CSF PhTau (*p* < 0.001) values increased with age. No difference was found between males and females, while ApoE4 carriers presented reduced CSF Aβ_42_, increased CSF PhTau and CSF Tau (all *p* < 0.001). Regarding physical function parameters, age subgroups presented no difference. Walking time was higher in females (*p* = 0.045) and grip strength was lower, as expected (*p* < 0.001). Grip strength was lower in ApoE ε4 carriers, as well (*p* = 0.034).

### 2.3. CSF Biomarkers in Relation to Physical Function Parameters

**Figure 1:**
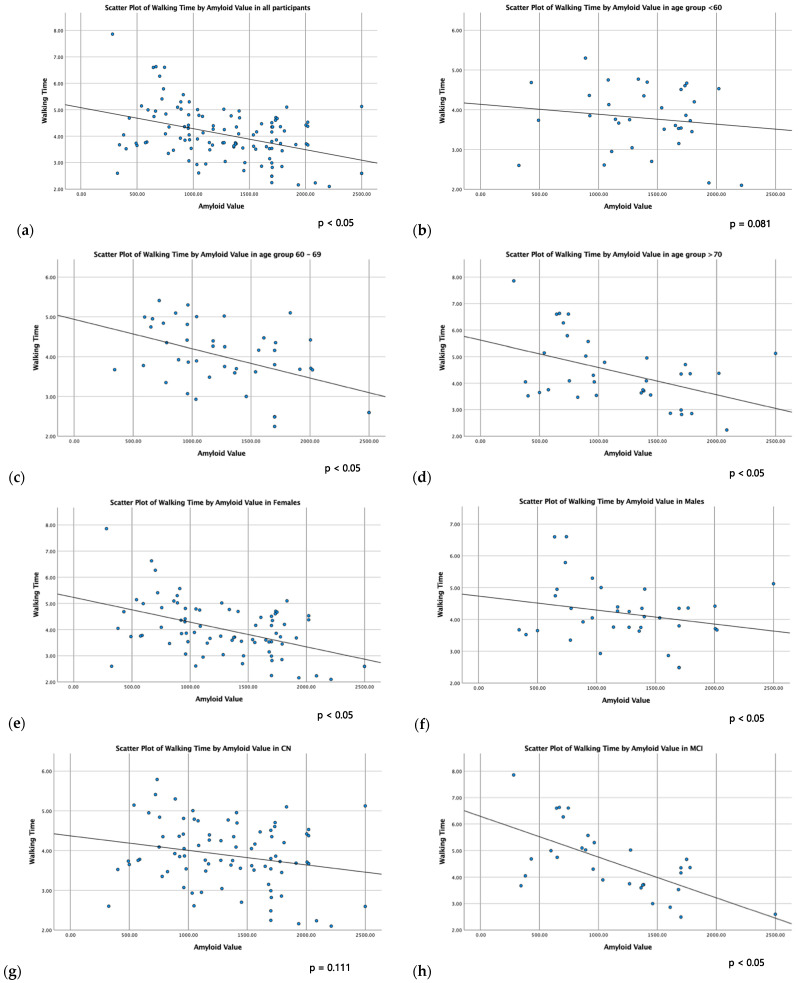
Scatter plots of linear correlation between walking time and CSF Aβ_42_ in all participants (**a**), age groups (**b**–**d**), sex groups (**e**,**f**) and cognitive groups (**g**,**h**).

Simple (unadjusted) linear correlations between physical function parameters and CSF biomarkers are presented in Table 3. Walking time was inversely related to CSF Aβ_42_ in all subgroups (*p* < 0.05), except CN individuals and participants younger than 60 years old. Regarding grip strength, subgroup analysis showed a negative correlation with CSF Tau and CSF PhTau in males (*p* < 0.05). Scatter plots of the linear correlation between walking time and CSF Aβ_42_ in all subgroups are presented in Figure 1.

**Figure 2:**
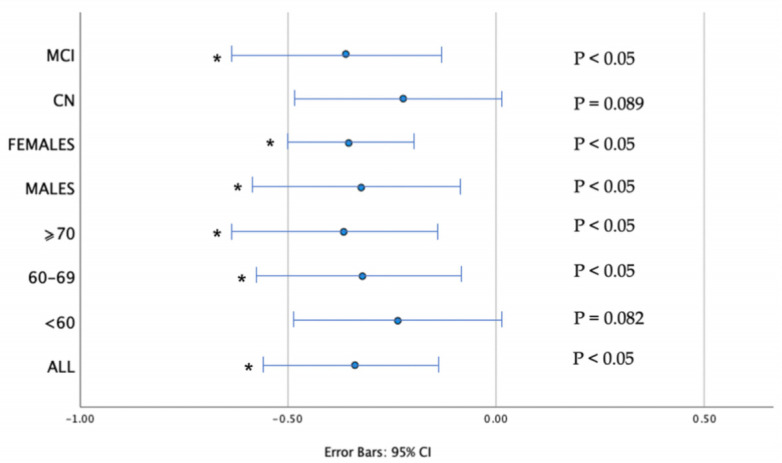
Regression Coefficient (b) of CSF Aβ_42_ οn walking time. Error bars represent 95% confidence intervals (CI). Results are corrected for age, sex, education, years, APOE status. Associations that remained significant after multiple regression analysis are indicated with an asterisk (all corrected *p* < 0.05).

Further statistical analysis of the effect of CSF biomarkers in physical function parameters was performed via multiple adjusted regression models (Table 4), in order to adjust for the main confounding factors. The results did not change after adjusting for age, education, years, sex and ApoE genotype. The regression coefficients of multiple regression models are shown in Figure 2.

## 3. Discussion

In our analysis walking time was inversely related to CSF Aβ_42_ in all ALBION participants. Subgroup analysis showed that this association concerned mainly individuals with MCI and participants older than 60 years old, a result which did not change after controlling for the confounding factors (such as age, sex, education and APOE status). This is the first study to examine CSF biomarkers in relation to physical function parameters in a relatively large number of participants, with a big proportion of them (68.7%, 112 in total) being CN.

To date, the literature had indicated contradictory results regarding the relation of Aβ_42_ and walking speed. To be precise, studies using CSF analysis did not provide statistically significant relations between gait speed and Aβ_42_ [21,22,23,24]. On the contrary, in PET studies, which are able to detect local amyloid deposition in different brain regions, Aβ_42_ was either negatively related to gait speed [25,26,27,28] or not related [29,30]. Our finding that Aβ_42_ and the equivalent of gait speed (walking time) were mainly related in participants with MCI, while in the subgroup of CN such a relation was not significant, is in accordance with Nadkarni et al. [25], who found that such a relation was weaker in CN participants, in relation to the whole study sample consisting of CN and MCI individuals. We found no sex-specific association, in contrast to two studies [26,31] which suggested that women may be more susceptible to the negative effects of AD pathology.

The mechanism of the association between amyloid deposition and gait speed is yet to be clarified. What we know is that brain amyloid deposition and gait performance are both related to cognition and share several risk factors, such as diet and smoking [32,33,34], cardiovascular disease [35,36] and expression of the APOE ε4 allele [34,37]. A potential explanation of such a relation was given in the context of the PET studies. Gait involves complex brain functioning and requires the coordination of motor as well as perceptual and cognitive processes [38]. It has been hypothesized that early amyloid deposition in motor-related brain regions, such as corticostriatal circuits, may play a key role in physical decline. Del Campo et al. [39] found that increased Aβ deposition in motor related regions (posterior and anterior putamen, occipital cortex, precuneus and anterior cingulate) was associated with decreased gait speed, enhancing the notion that amyloid pathology may cause neuronal dysfunction and a neurotoxic effect either directly or indirectly by accelerating Tau deposition and, consequently, neurodegeneration [40].

Nevertheless, Tau, which is a measure related to neurodegeneration in general and not particularly AD, was not measured in relevant PET studies, but results are available from the four aforementioned CSF studies [21,22,23,24]. In accordance with our findings, Tau was not related to gait speed.

Concerning handgrip strength, although it has already been recognized as a factor of physical decline and has been related to cognition [11], data concerning its relation to AD biomarkers are limited. Yoon et al. [34] concluded that amyloid in all brain regions in PET was linked significantly to low grip strength, however Legdeur et al. [41] did not find any significant relation. Our analysis showed a relation between grip strength and CSF Tau, CSF PhTau only in males, however, the result was rendered not significant after adjustment for confounders. Hence, more research is needed to examine whether any significant relation exists.

Our findings expand existing knowledge concerning the correlation between two factors related to cognitive performance. Walking speed is an important indicator of physical performance of the lower extremities, while CSF amyloid has already been recognized as a biomarker of the AD continuum. If our findings are validated by additional research, involving longitudinal study designs that might enable cause-effect conclusions, such an association may place emphasis on maintaining physical performance as a preventive and therapeutic strategy against the progression of cognitive decline and dementia.

Our study presents several limitations that need to be considered for the interpretation of the results. First of all, this was a cross-sectional analysis which could not detect longitudinal differences in physical function. Longitudinal data is being collected (so far, up to five years for a few participants) allowing future estimations of clinical progression and physical decline over time. Additionally, selection bias existed, mainly due to the fact that some participants were self-referred if concerned about their memory or had a positive family history of late-onset AD dementia. Hence, a proportion of participants might have been evaluated too early (in a relatively young age) to obtain significant results, especially in physical function parameters which are strongly correlated with aging and, thus, our results may have relatively low generalizability. Our study sample was of a broad age range (40–75); thus, subgroup sensitivity analysis was performed to reduce the influence of the specific limitation. Furthermore, we could not check various gait parameters, apart from walking speed, such as fast walking and dual-task walking. New, recently proposed tools for motion assessment like the inertial measurement unit (IMU) [42] could provide more specific and sensitive results.

Regarding the strengths of the present analysis, automated methods for CSF biomarkers assessment were used, which seem to be concordant with amyloid PET scan imaging [43], in relation to previously used assays. Clinical evaluation was carried out by clinicians with subspecialty training and considerable experience in the cognitive disorders field. In addition, our study sample was larger than previous CSF studies examining physical function parameters, although smaller in relation to several PET studies. It also included a high proportion of CN individuals, who had not been studied in past research. All participants had a negative medical history of brain pathology, hence gait disturbances could not be attributed to other neurological disorders.

## 4. Materials and Methods

### 4.1. Participants and Study Design

Data for the analysis were drawn from the ALBION (Aiginition Longitudinal Biomarker Investigation of Neurodegeneration) study. The sample consisted of individuals aged ≥ 40 years, referred by other specialists or self-referred to the cognitive disorders’ outpatient clinic of Aiginition, Athens, Greece [44]. Only cognitively normal (CN) individuals or individuals with MCI at baseline, based on established diagnostic criteria, were included in the study [45].

Patients with a diagnosis of dementia were not included in the study, as well as patients with neurological, psychiatric, or medical conditions associated with a high risk of cognitive impairment or dementia (including but not limited to Parkinson’s disease, multiple sclerosis, Huntington’s disease, Down syndrome, active alcohol or drug abuse or major psychiatric conditions such as major depressive disorder, schizophrenia and bipolar disorder). The present analysis contains ALBION participants in their first evaluation consisting of a neuropsychological evaluation through clinical examination and relevant questionnaires, lumbar puncture, blood sampling and physical function parameters’ assessment, all performed on the same day. A total of 163 individuals were included in the analysis. More details about the study design and data collection procedure can be found elsewhere [46].

### 4.2. Diagnostic Procedures

#### 4.2.1. Neurological and Neuropsychological Evaluation

Clinical diagnosis was established by a specialist neurologist after an extensive standardized neuropsychological assessment. Detailed information regarding medical and family history, lifestyle and demographics were collected by certified neurologists, licensed neuropsychologists, and registered dieticians. All participants received a thorough neurological and neuropsychological assessment through structured questionnaires, clinical examination and neuropsychological tests. Global cognition was assessed using the Mini Mental State Examination (MMSE) [47] as well as the Revised Addenbrooke’s Cognitive Examination (ACE) [48] and diagnosis of MCI was established using standard criteria [49]. MCI and MCI subtypes (memory, executive speed, visual, spatial, language and combinations) were assigned when participants had subjective memory complaints and objective impairment in at least one cognitive domain, but preserved activities of daily living.

#### 4.2.2. Cerebrospinal Fluid (CSF) Analysis

CSF quantitative analysis was available on all participants. The procedure of the lumbar puncture and the collection and storage of cerebrospinal fluid (CSF) was conducted according to international guidelines [50]. For the purposes of the ALBION study, CSF was primarily processed for biomarkers such as amyloid Aβ_42_, Tau and PhTau which are indicative of AD. CSF samples were analyzed using automated Elecsys^®^ assays (Roche Diagnostics, Maroussi, Greece). The provided reference ranges for a positive result were as follows: Aβ_42_ ≤ 1000 pg/mL, Tau > 300 pg/mL and PhTau > 27 pg/mL.

#### 4.2.3. Blood Analysis—ApoE Genotyping

APOE genotyping was performed in genomic DNA extracted from blood buffy coat, using Qiamp DNA Blood Mini Kits (Qiagen, Venlo, The Netherlands). The method used for genotyping was polymerase chain reaction-DNA sequencing, carried out with a LightCycler 2 (Roche Diagnostics GmbH) and using the LightMix TIB MOLBIOL (Berlin, Germany) reactors. Genotyped data were anonymized and treated in a blinded manner for the clinical information of participants.

#### 4.2.4. Physical Function Parameters Assessment

Physical function parameters were assessed during the first evaluation, a few minutes before performing the lumbar puncture. Physical performance of the lower extremities was assessed with the 4 m measured walk. After a demonstration, participants were instructed to walk a 4 m distance at their normal gait speed. Each participant had two timed trials to complete the 4 m distance. For each effort, we recorded the time in seconds needed to complete the course with a stopwatch.

Physical strength of the upper extremities was estimated using a handgrip electronic dynamometer (model MG4800, Marsden, Rotherham, UK). Maximum isometric strength of the dominant hand was recorded three times with an interval of 30 s between each effort. The participant was in the standing position and the elbow at a 90° angle without touching the body.

### 4.3. Statistical Analyses

All analyses were performed using SPSS for Windows Version 28.0 (IBM Corp, Armonk, NY, USA). Descriptive statistics were calculated for participants’ characteristics (age, sex, education, family history of dementia), cognitive function (relevant MMSE, ACE scores), and physical function parameters (walking time, handgrip strength). Participants were separated in two groups according to their cognitive status [cognitively normal (CN) and MCI]. Continuous variables were expressed as mean values and standard deviations, while categorical variables were referred to as frequencies and percentages. Statistical significance was set at *p* < 0.05 a priori.

For statistical purposes, walking time and handgrip strength measurements were converted to an average score from the individual trials. ApoE genotyping data were dichotomized into ε4 carriers (defined as presence of one or two ε4 alleles) and non-carriers. CSF biomarkers were treated as continuous variables, using the exact CSF Aβ_42_, Tau and PhTau values. To compare continuous variables such as age, education, cognitive scores, and physical function parameters between the two groups, we used analysis of variance (ANOVA). To compare CSF biomarkers’ values we used the Wilcoxon Rank Sum Test. For categorical variables (such as sex, family history of dementia and ApoE status) we used the Fisher exact test.

Subgroup analysis was performed for CSF biomarkers as well as physical function parameters. Age subgroups (<65 and ≥65 years old) were formed according to the median (which was 64.6 years). Sex groups included males and females, while ApoE status was separated in ε4 carriers and non-carriers. Walking time and handgrip strength were compared via ANOVA in subgroups, while CSF biomarkers were compared via Wilcoxon Rank Sum Test.

In order to examine the association between physical function parameters and CSF biomarkers, we first performed linear correlational analyses between each parameter and each biomarker, using Spearman’s rank correlation coefficient since some variables had a non-parametric distribution. The above-mentioned subgroups were used, as well as additional age subgroups (<60, 60–69, ≥70) which were created for sensitivity analysis.

We then proceeded with multivariate linear regression analyses, in order to adjust for the main confounding factors of such correlations. Walking time and handgrip strength were treated as dependent variables and we performed separate models for each CSF biomarker (CSF Aβ_42_, CSF Tau and CSF PhTau independent variables) in all subgroups. All models were adjusted for age, sex, education and ApoE status.

## 5. Conclusions

Cerebrospinal fluid biomarkers of the AD continuum precede clinical manifestations of dementia, as they are present in pre-clinical stages as well. In the absence of effective treatment, research has focused on the early detection of increased risk and the prevention of AD dementia. Lifestyle factors seem to prevent clinical progression, including physical activity which seems really promising. Our analysis highlighted that walking time is inversely related to CSF Aβ_42_ in individuals in the course of the AD continuum, mainly those older than 60 years old and participants with MCI. The detailed mechanisms underlying this association are yet to be determined, especially in the context of whether amyloid accumulation influences gait performance per se, or as one of many aspects of neurodegeneration. Our study expands knowledge concerning the relation between walking performance and CSF amyloid, an already recognized AD biomarker, contributing significantly to the existing literature, and opening new perspectives for exploration in research concerning mobility in patients in the AD continuum and the possible identification of new biomarkers.

## Figures and Tables

**Table 1:** Baseline participants’ characteristics using clinical diagnosis.

	All (n = 163)	CN ^1^ (n = 112)	MCI ^2^ (n = 51)	*p*-Value
Sex, female (%)	109 (66.9)	76 (67.9)	36 (70.6)	0.722
Age, y, mean ± SD ^3^	64.3 ± 9.2	62.8 ± 9.2	67.6 ± 8.2	0.002
Education, y ^4^, Mean ± SD	13.3 ± 3.8	13.8 ± 3.7	12.3 ± 4.1	0.016
Family History of Dementia, n (%)	75 (46.0)	53 (47.3)	22 (43.1)	0.734
MMSE ^5^, Mean ± SD	28.1 ± 1.8	28.9 ± 1.2	26.4 ± 1.8	**<0.001**
ACE ^6^ score, Mean ± SD	90.0 ± 7.7	93.6 ± 4.5	81.9 ± 7.1	**<0.001**
ApoE_4_ carrier, positive (%)	44 (28.0) n = 157	22 (20.6) n = 107	22 (44.0) n = 50	**0.004**
Walking Time, s ^7^, Mean ± SD	4.73 ± 1.76 (n = 114)	4.33 ± 1.04 (n = 81)	5.82 ± 2.65 (n = 33)	**<0.001**
Grip Strength, kg ^8^, Mean ± SD	25.7 ± 8.9 (n = 143)	26.8 ± 9.3 (n = 98)	23.3 ± 7.8 (n = 45)	**0.032**
CSF Aβ_42_, Mean ± SD	1161.6 ± 529.4	1240.0 ± 508.5	989.2 ± 538.3	**0.005**
CSF Tau, Mean ± SD	230.4 ± 172.5	218.0 ± 196.9	257.9 ± 96.5	0.171
CSF PhTau, Mean ± SD	19.2 ± 9.9	17.2 ± 8.9	23.6 ± 10.7	**<0.001**

^1^ Cognitively Normal, ^2^ Mild Cognitive Impairment, ^3^ Standard Deviation, ^4^ years, ^5^ Mini Mental State Examination, ^6^ Addenbrooke’s Cognitive Examination, ^7^ seconds, ^8^ kilograms. Bold values indicate statistically significant difference between the two groups.

**Table 2:** CSF biomarkers and physical function parameters in different groups.

	Age Groups		Sex Groups		ApoE4 Groups	
	<65	≥65	Males	Females	Carriers	Non-Carriers
N = 163	78	85	54	109	44	113
**CSF Aβ_42_**, mean ± SD ^1^, *p*-Value	1278.0 ± 478.6	1054.8 ± 553.5	1122.5 ± 536.3	1181.0 ± 527.3	882.3 ± 428.8	1276.8 ± 524.1
	**0.007**		0.508		**<0.001**	
**CSF Tau**, mean ± SD, *p*-Value	193.9 ± 96.2	264.0 ± 215.8	235.3 ± 99.3	228.0 ± 199.5	324.2 ± 281.2	196.2 ± 88.4
	**0.009**		0.801		**<0.001**	
**CSF PhTau**, mean ± SD, *p*-Value	16.3 ± 9.4	21.9 ± 9.7	21.0 ± 9.1	18.3 ± 10.3	26.0 ± 11.9	16.7 ± 7.9
	**<0.001**		0.801		**<0.001**	
N = 114	56	58	36	78	25	83
**Walking Time**, s ^2^, mean ± SD, *p*-Value	4.53 ± 2.13	4.93 ± 1.29	4.25 ± 0.90	4.96 ± 2.00	4.80 ± 1.25	4.76 ± 1.91
	0.220		**0.045**		0.884	
N = 143	69	74	43	100	38	99
**Grip Strength**, kg ^3^, mean ± SD, *p*-Value	26.0 ± 9.0	25.4 ± 8.9	35.8 ± 8.2	21.4 ± 4.7	22.9 ± 8.2	26.6 ± 9.0
	0.348		**<0.001**		**0.034**	

^1^ Standard Deviation, ^2^ seconds, ^3^ kilograms. Bold values indicate statistically significant difference between the two groups.

**Table 3:** Simple linear correlations between walking time, grip strength and CSF AD biomarkers.

**Walking Time**	**All**	**Age Groups**		**Age Groups**			**Sex Groups**		**Clinical Groups**	
		**<65**	**≥65**	**<60**	**60–69**	**≥70**	**Males**	**Females**	**CN ^1^**	**MCI ^2^**
N=	114	56	58	37	44	36	36	78	81	33
**CSF Aβ_42_** r_s_ ^3^ (*p*)	−0.288 **(<0.05)**	−0.241 **(<0.05)**	−0.318 **(<0.05)**	−0.183 (0.081)	−0.261 **(<0.05)**	−0.312 **(<0.05)**	−0.281 **(<0.05)**	−0.295 **(<0.05)**	−0.151 (0.111)	−0.343 **(<0.05)**
**CSF Tau** r_s_ (*p*)	0.111 (0.091)	0.039 (0.773)	0.077 (0.565)	0.052 (0.724)	0.031 (0.818)	0.066 (0.663)	0.061 (0.663)	0.148 (0.124)	0.029 (0.764)	0.274 (0.052)
**CSF PhTau** r_s_ (*p*)	0.115 (0.143)	0.063 (0.643)	0.017 (0.901)	0.015 (0.836)	0.007 (0.957)	0.087 (0.532)	0.119 (0.390)	0.127 (0.190)	0.019 (0.845)	0.215 (0.130)
**Grip Strength**	**All**	**Age Groups**		**Age Groups**			**Sex Groups**		**Clinical Groups**	
		**<65**	**≥65**	**<60**	**60–69**	**≥70**	**Males**	**Females**	**CN**	**MCI**
N=	143	69	74	46	55	57	43	100	98	45
**CSF Aβ_42_** r_s_ (*p*)	0.044 (0.597)	0.062 (0.613)	0.049 (0.681)	0.020 (0.989)	0.051 (0.716)	0.061 (0.687)	0.037 (0.815)	0.051 (0.612)	0.128 (0.180)	0.079 (0.434)
**CSF Tau** r_s_ (*p*)	−0.060 (0.472)	−0.051 (0.677)	−0.223 (0.056)	−0.048 (0.756)	−0.060 (0.666)	−0.123 (0.416)	−0.362 **(<0.05)**	−0.058 (0.566)	−0.085 (0.371)	−0.053 (0.605)
**CSF PhTau** r_s_ (*p*)	−0.103 (0.221)	−0.088 (0.471)	−0.180 (0.127)	−0.03 (0.987)	−0.09 (0.948)	−0.08 (0.599)	−0.432 **(<0.05)**	−0.116 (0.249)	−0.118 (0.217)	−0.116 (0.254)

^1^ Cognitively Normal, ^2^ Mild Cognitive Impairment, ^3^ Spearman’s correlation coefficient. Bold values indicate statistically significant results.

**Table 4:** Association between walking time, grip strength and CSF AD biomarkers adjusted for age, education, years, sex and ApoE via multivariate linear regression models.

**Walking Time**	**All**	**Age Groups**		**Age Groups**			**Sex Groups**		**Clinical Groups**	
		**<65**	**≥65**	**<60**	**60–69**	**≥70**	**Males**	**Females**	**CN ^1^**	**MCI ^2^**
N=	114	56	58	37	44	36	36	78	81	33
**CSF Aβ_42_** b ^3^ (*p*)	−0.346 **(<0.05)**	−0.332 **(<0.05)**	−0.365 **(<0.05)**	−0.236 (0.082)	−0.323 **(<0.05)**	−0.360 **(<0.05)**	−0.342 **(<0.05)**	−0.365 **(<0.05)**	−0.223 (0.089)	−0.387 **(<0.05)**
**CSF Tau** b (*p*)	0.031 (0.759)	0.012 (0.942)	0.015 (0.852))	0.065 (0.757)	0.143 (0.371)	0.067 (0.775)	0.061 (0.663)	0.054 (0.668)	0.049 (0.664)	0.163 (0.253)
**CSF PhTau** b (*p*)	0.031 (0.787)	0.047 (0.778)	0.073 (0.633	0.099 (0.623)	0.074 (0.638)	0.089 (0.720)	0.119 (0.390)	0.043 (0.769)	0.028 (0.746)	0.118 (0.331)
**Grip Strength**	**All**	**Age Groups**		**Age Groups**			**Sex Groups**		**Clinical Groups**	
		**<65**	**≥65**	**<60**	**60–69**	**≥70**	**Males**	**Females**	**CN**	**MCI**
N=	143	69	74	46	55	57	43	100	98	45
**CSF Aβ_42_** b (*p*)	0.128 (0.180)	0.068 (0.368)	0.70 (0.606)	0.144 (0.334)	0.157 (0.250)	0.075 (0.561)	0.033 (0.813)	0.062 (0.527)	0.155 (0.107)	0.203 (0.152)
**CSF Tau** b (*p*)	−0.011 (0.853)	−0.031 (0.733)	−0.055 (0.670)	−0.130 (0.364)	−0.044 (0.582)	−0.253 (0.206)	−0.246 *(0.125)*	−0.089 (0.396)	−0.013 (0.841)	−0.016 (0.899)
**CSF PhTau** b (*p*)	−0.073 (0.246)	−0.018 (0.84)	−0.128 (0.132)	−0.113 (0.427)	−0.053 (0.509)	−0.130 (0.296)	−0.274 (0.098)	−0.05 (0.968)	−0.115 (0.088)	−0.070 (0.572)

^1^ Cognitively Normal, ^2^ Mild Cognitive Impairment, ^3^ Regression Coefficient. Bold values indicate statistically significant results.

## Data Availability

The data that support the findings of this study are available from the study’s principal investigator, NS, upon reasonable request.
